# Identifying kinematic biomarkers of the dystrophic phenotype in a zebrafish model of Duchenne muscular dystrophy

**DOI:** 10.1186/s13395-025-00382-6

**Published:** 2025-06-20

**Authors:** Jeffrey J. Widrick, Matthias R. Lambert, Felipe de Souza Leite, Youngsook Lucy Jung, Junseok Park, James R. Conner, Eunjung Alice Lee, Alan H. Beggs, Louis M. Kunkel

**Affiliations:** 1 Division of Genetics and Genomics, Dept. of Pediatrics, Boston Children’s Hospital, Boston, MA USA; 2 Harvard Medical School, Boston, MA USA; 3 The Manton Center for Orphan Disease Research, Boston Children’s Hospital, Boston, MA USA

**Keywords:** Duchenne muscular dystrophy, Kinematics, Swimming, Mobility, DeepLabCut, Markerless motion capture, *Sapje*, *Sapje-like*

## Abstract

**Background:**

Dystrophin-deficient zebrafish larvae are a small, genetically tractable vertebrate model of Duchenne muscular dystrophy that is well suited for early-stage therapeutic development. However, current approaches for evaluating their mobility, a physiologically relevant therapeutic outcome, yield data of low resolution and high variability that provides minimal insight into potential mechanisms responsible for their abnormal locomotion.

**Methods:**

To address these issues, we used high speed videography and deep learning-based markerless motion capture to quantify escape response (ER) swimming kinematics of two dystrophic zebrafish strains (*sapje* and *sapje-like*). Each ER was partitioned into an initiating C-start, a subsequent power stroke, and a final burst of undulatory swimming activity.

**Results:**

Markerless motion capture provided repeatable, high precision estimates of swimming kinematics. Random forest and support vector machine prediction models identified overall ER distance and peak speed, the instantaneous speed conferred by the power stroke, and the average speed and distance covered during burst swimming as the most predictive biomarkers for differentiating dystrophic from wild-type larvae. For each of these predictors, mutant and wild-type larvae differed markedly with effect sizes ranging from 2.4 to 3.7 standard deviations. To identify mechanisms underlying these performance deficits, we evaluated the amplitude and frequency of propulsive tail movements. There was little evidence that tail stroke amplitude was affected by the absence of dystrophin. Instead, temporal aspects of tail kinematics, including tail maximal angular velocity during the C-start and power stroke and tail stroke frequency during burst swimming, were slowed in mutants. In fact, tail kinematics were as effective as direct, non-survival in vitro assessments of tail muscle contractility in differentiating mutant from wild-type larvae.

**Conclusions:**

ER kinematics can be used as precise and physiologically relevant biomarkers of the dystrophic phenotype, may serve as non-lethal proxies for skeletal muscle dysfunction, and reveal new insights into why mobility is impaired in the absence of dystrophin. The approach outlined here opens new possibilities for the design and interpretation of studies using zebrafish to model movement disorders.

**Supplementary Information:**

The online version contains supplementary material available at 10.1186/s13395-025-00382-6.

## Background

Duchenne muscular dystrophy (DMD), a progressive pediatric neuromuscular disorder caused by loss of function mutations to the X-linked dystrophin gene [1], is characterized by delays in motor development, excessive muscle weakness, impaired ambulation, and eventual cardio-respiratory failure [2]. Developing therapies and cures for DMD has historically relied on small mammalian models, principally the *mdx* mouse. Dystrophin-deficient zebrafish larvae, such as *sapje* [3] and *sapje-like* [4], have emerged as alternatives or compliments to mammalian models due to their small size, rapid *ex utero* development, body transparency, permeability to small molecules, and genetic tractability. These features make zebrafish larvae ideal for probing the relationship between vertebrate genotype and disease phenotype and facilitate experimental approaches that would be difficult to conduct with other vertebrate DMD models, such as large-scale screens of potential therapeutics [5–7].

High resolution approaches for quantifying phenotype are critical tools for the discovery and development of therapeutic compounds and treatments [8]. However, there is growing concern in the broader biomedical research community that advances in genetics and molecular biology are outpacing the methods available for evaluating phenotype and that this imbalance may eventually impede scientific progress [9, 10]. This is particularly important for zebrafish models of muscle disease where arguably their most physiologically relevant phenotype is mobility, yet current approaches for quantifying motor activity typically use low temporal and spatial resolution measurements of spontaneous motor activity [11]. Data collected in this manner are often characterized by large intra- and inter-larval variability while providing limited insight into mechanisms underlying impaired mobility.

The present project was designed to address these issues. Zebrafish larvae display a range of motor behaviors enabling them to hunt, capture prey, and avoid perceived threats [12–15]. We chose to study their startle or escape response, a stereotypic behavior mediated by an all-or-none activation of the musculature of the tail and trunk that produces a brief burst of coordinated, high-intensity swimming activity [16–18].

Because escape responses occur on a millisecond (ms) time scale, we utilized high speed videography to obtain high temporal resolution images of escape response movement of two different strains of dystrophin-deficient zebrafish larvae, *sapje* and *sapje-like* [3, 4]. We then developed a markerless motion capture approach that extracted keypoint coordinates from the videos and enabled us to model each escaping larvae as a linked segment system. This approach revealed new insight into how the absence of dystrophin impacts vertebrate locomotion and identified statistically powerful, physiologically relevant, non-lethal biomarkers of the dystrophic phenotype.

## Methods

### Zebrafish

Fertilized zebrafish eggs, obtained from matings of heterozygous adults, were collected and cultured in 100 cm petri dishes at 28.5 °C with a 14 h:10 h light:dark cycle. All adult zebrafish and larvae were maintained in a fish facility overseen by the Aquatic Resources Program at Boston Children’s Hospital. Data on water quality, environmental conditions, diet, and other aspects of husbandry are available on protocols.io (10.17504/protocols.io.br4mm8u6).

### Birefringence assay

A non-lethal birefringence assay was used to classify larvae as affected or unaffected as previously described [19, 20]. Briefly, lightly anesthetized larvae were aligned between two glass polarizing filters, viewed with a stereo-microscope, and classified as unaffected if the tail and trunk musculature appeared bright and well-organized or affected of the muscle displayed gaps, breaks, or loss of birefringence. Affected and unaffected larvae were then transferred into 48 well plates, one larvae per well, and returned to the incubator until further study.

### Experimental setup

Escape responses were evaluated in a rectangular arena formed by laser cutting a 20 mm × 30 mm opening in a 30 mm × 40 mm piece of acrylic. The arena was attached to the inside surface of a water-jacketed preparatory tissue dish (Radnoti, model 158,401) with aquarium grade silicon. Water from a temperature-controlled bath circulated through the jacket and beneath the arena. The arena contained fish water to a depth of ≈ 3 mm at the center in order to minimize movements in the z-plane. A micro thermocouple confirmed that the fish water temperature was maintained at 25 °C throughout data collection. An LED array and diffuser illuminated the arena from below.

### Escape responses

A single larva was transferred into the arena and given 5 min to temperature equilibrate. Escape responses were elicited by a 1 ms electric field pulse [21] generated by a constant current muscle stimulator (Aurora Scientific, model 701) and delivered to platinum electrodes aligned along opposite walls of the arena. In preliminary studies, we established the current that consistently elicited an escape response and used this current for all subsequent trials.

Each larva was subjected to multiple trials until we had three acceptable escape responses (see quality control criteria below). A minimum of 60 s separated successive trials which is four times the inter-escape response period previously used to prevent habituation [12].

### High speed videography

Videos (1280 × 864 pixels) were collected at 1000 frames/s using a monochrome high speed camera (Edgertronic SC2) positioned approximately 9 cm above the arena. A Nikon 50 mm f1.8D lens fitted with a 10X close-up lens produced a field of view that was roughly the same dimensions as the arena. Distance was calibrated each day of data collection and ranged from 44–45 pixels per mm.

A manually triggered, opto-isolated circuit was constructed to coordinate the escape response stimulus with the video recording. When triggered, the circuit opened the camera shutter but delayed the escape response stimulus by 10 ms producing 10 pre-stimulus frames for each video.

### Markerless pose estimation

The open-source machine learning toolkit DeepLabCut (version 2.2rc3) was used to create a deep neural network for estimation of larval pose [22, 23]. To develop the neural network, we collected escape responses of 10 wild-type AB larvae (6 pdf). A kmeans algorithm selected 25 frames from each video that encompassed a diversity of poses. Each of the 250 frames were manually annotated with the following keypoints: TS, tip of the snout; S1, anterior aspect of the swim bladder; S2, posterior aspect of the swim bladder; T4 tip of trunk or tail; T2, point midway between S2 and T4; T3, point midway between T2 and T4; T1, point midway between S2 and T2. The keypoints defined six body segments: head, swim bladder, tail1, tail2, tail3, tail4. The keypoints were chosen so that we could quantify the length of the larva’s body, track its approximate center of mass, and model the curvature of its body.

The neural network was initially trained using ResNet50 architecture for 7.5 × 10^5^ iterations using an 80% training to 20% testing split of the annotated dataset. Likelihood values were sometimes low during C-starts, when the tip of the tail was aligned very closely to, or even temporally occluded by, the head. To refine the network, we identified escape responses from six wild-type larvae where this occurred (3–8 frames per video). Keypoints with low likelihood scores (< 0.98) were either manually re-labeled or deleted (if occluded). These 28 newly annotated frames were merged with the original 250 annotated frames, the new data set was split into training (80%) and test (20%) sub-sets, and the neural network trained for 10^6^ iterations.

Network training was conducted on a Dell Precision 3640 desktop computer (Intel Core i7-10700 K, 8 Core, 32 GB RAM, Ubuntu version 20.04 LTS) equipped with a NVIDIA GeForce RTX 3080 graphical processing unit. With this configuration, the 10^6^ iterations used to train the neural network were completed in about 12 h. Escape response videos were analyzed on the same hardware, requiring about 5 s per trial. The DeepLabCut neural network developed for estimating larva pose from an escape response video is available at https://github.com/jjwidrick/danio-ER-DNN. 

### Kinematic analysis

Custom scripts written in R [24] were used to calculate morphological and kinematic variables from the DNN output. These scripts have been incorporated into an R package named “daniomotion” which is available at https://github.com/jjwidrick/daniomotion. Tail segment angle data were smoothed using locally estimated scatterplot smoothing (LOESS, span = 0.10) prior to analysis. Tail curvature was defined as the sum of the four individual trunk segment angles. Tail rotation direction was standardized between larvae by defining positive rotation as the direction of the first major bend of the tail (the C-start). Tail curvature angular velocity was calculated using the central finite difference equation [25]. The initiation of movement was determined as the post-stimulus frame where tail curvature exceeded the preceding frame by 2%.

Linear kinematics were based on the movement of the larva’s center of mass (COM). Larval COM is reported to fall at various locations between the anterior and posterior edges of the swim bladder [14, 15, 26, 27]. For the purposes of this study, we used keypoint S2 to represent the COM [14]. Distance was defined as the length between S2 on consecutive frames. Displacement was defined as the vector between S2 position at movement time zero and the current S2 position. Distance and displacement were calculated from unfiltered data.

The instantaneous speed of S2 was determined as the first derivative of distance with respect to time using the central difference method. The acceleration of S2 was determined as the second derivative of displacement with respect to time. Instantaneous speed oscillates due to the undulatory nature of swimming. In order to increase consistency in determining the exit speed from each stage as well as the overall peak instantaneous speed, the raw speed response was smoothed using LOESS (span = 0.7).

Linear kinematics were normalized to larval body length (BL). Body length was calculated as the sum of the 6 body segments, averaged across the pre-movement frames.

### Stage-specific kinematics

We calculated performance variables that took into account the entire escape response (overall distance, displacement, peak instantaneous speed, and peak acceleration). We also partitioned the escape response into three stages [28] and determined stage specific kinematics. The first change in tail curvature sign delineated the transition from the stage 1 C-start to the stage 2 power stroke. The next change in sign indicated the end of the power stroke and the beginning of stage 3 burst swimming. Some larvae had a small counter tail bend that preceded the much larger C-start. These bends were ignored in analysis [29].

We used successive extremes in tail curvature to define a tail stroke (two sequential tail strokes equals one tail beat cycle). Stage 1 and 2 consist of single tail strokes. Stage 3 consists of multiple strokes and we choose to carry the stroke nomenclature through stage 3 rather than convert strokes to tail beats. The number of stage 3 tail strokes varied between larvae. Therefore, for stage 3 we calculated variables that encompassed the entire stage (total stage duration, total stage distance, total stage displacement, and average stage speed) as well as variables that were based on the number of full strokes completed by the larvae, ignoring any partial strokes at the end of the stage. These variables were the average number of tail strokes, the average distance covered/tail stroke, the average tail stroke peak to peak amplitude, and the average tail stroke frequency.

### Muscle contractility

A subset of *sapje* strain larvae that had been subjected to ER analysis were subsequently used for assessment of muscle contractility as previously described [30]. Briefly, a larva was euthanized and one end of the tail was attached to the output tube of an isometric force transducer. Attachment was made at the gastrointestinal opening. This attachment point was easy to replicate as the 10–0 silk thread used for fastening the preparation was guided to this location by the notch formed at the intersection of the ventral and dorsal fin folds. This consistency in attachment enabled valid absolute force comparisons between preparations. The other end of the preparation was attached to an immobile titanium wire at a location that was approximately 2 mm distal to the GI opening.

Tail muscle preparations were studied in a bicarbonate buffer (equilibrated with 95% O_2_, 5% CO_2_) that was maintained at the same temperature as the escape response trials (25 °C). A muscle twitch was induced using a single supra-maximal square wave pulse, 200 µs in duration, that was delivered to platinum electrodes flanking the preparation. The length of the preparation was adjusted to maximize twitch force. Contraction time, half-relaxation time, and the maximal rates of tension development and relaxation were calculated from the peak twitch force records [30].

### Genotyping

Larvae were euthanized after completion of their escape trials. Genomic DNA was extracted from the heads and used as a PCR template. The *sapje* and *sapje-like* primer sets have been previously described [31]. Sanger sequencing was conducted at the Molecular Genetics Core Facility at Boston Children’s Hospital.

### Quality control

Raw videos were analyzed by the DNN without any pre-processing. Each escape response trial had to satisfy the following criteria in order to be included in further analysis. First, larvae had to remain stationary during the 10 ms pre-stimulus period. Second, the trial had to be accurately tracked by the neural network. Poor tracking was easily identified by a string of likelihood values < 0.98 coupled with erratic trunk curvature plots. Third, the video had to capture sufficient frames to enable us to analyze 60 ms of movement. Finally, only trials where there was agreement between fish phenotype (birefringence) and genotype (Sanger sequencing) were included in the final analysis, i.e. all affected fish genotyped as −/− and all unaffected fish as either +/+ or +/−.

### Statistical analysis

We evaluated 678 escape responses recorded from 49 *sapje* mutants and 73 wild-type siblings (29 +/+, 44 +/−) and 51 *sapje-like* mutants and 53 wild-type siblings (16 +/+, 37 +/−). These larvae were obtained from 7 and 4 matings of groups of adult *sapje* and *sapje-like* fish, respectively. Approximately 40% of the *sapje* strain larvae were subsequently used in the muscle physiology experiments (11 +/+, 20 +/−, 17 −/−). Unless otherwise noted, all results are based on these sample sizes.

Point estimates of effect sizes and variability were calculated using a bias-corrected-and-accelerated bootstrap approach. Standardized effect sizes were calculated using Hedges’s *g* with bias-corrected-and-accelerated intervals. Both methods used 5000 resamples and 99% confidence intervals.

To classify mutant (−/−) and wild-type (+/+ and +/−) groups, we employed a random forest model. All variables, regardless of stages, were used for modeling. For constructing trees, 2/3 of the data were used for training, and the remaining data were used for the out-of-bag calculation. To identify the important variables, the means of GINI importance were measured. To validate the random forest model, we employed a linear support vector machine (SVM) model with various values of C (ranging from 0.00 to 5.00) to gain the most accurate model, allocating 70% of the data for training and 30% for testing. Our validation process included conducting a tenfold cross-validation.

Statistical analysis was conducted with R version 4.4.1 [24] and the following packages: bootES [32], factoextra [33], FactoMineR [34], randomForest [35], ROCR [36], e1071 [37], kernlab [38], ROCit [39].

## Results

### Capturing escape responses

Initial studies were conducted on *sapje* (*sap*^*ta222a*^) zebrafish which have a nonsense mutation in exon 4 of the dystrophin gene [3]. To control for any allele-specific effects [40], we studied a second dystrophin mutant, *sapje-like* (*sap*^*cl100*^), which is characterized by a mutation in the splice donor site of exon 62 [4]. Both of these strains lack the full length skeletal muscle dystrophin isoform, which when it occurs in humans is the cause of Duchenne muscular dystrophy. However, because of downstream alternative start sites, *sapje* expresses five short dystrophin isoforms (Dp260, Dp140, Dp116, Dp71, and Dp40) while *sapje-like* is expected to only express the two shortest [41].

**Fig. 1:**
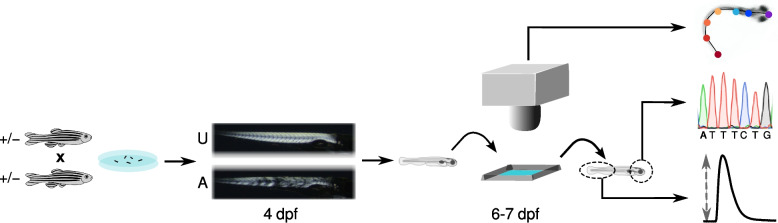
Overview of the experimental design. Larvae were obtained by mating heterozygous adults. At 4 day post-fertilization (dpf) a birefringence assay was used to classify larvae as having either an unaffected (**U**) or an affected (**A**) muscle phenotype. On 6 and 7 dpf, larvae were transferred one at a time into a small arena containing fish water where three escape responses, induced by a 1 ms electrical field pulse, were recorded at 1000 frames/s. Videos were used to evaluate linear and angular kinematics. Larvae were euthanized after their escape responses and DNA extracted from the head was used to confirm larval genotype. In a subset of post-escape response larvae, a portion of the tail was assayed for twitch contractile properties

Because mutants have a median lifespan of only 30 days [3, 20, 31], larvae for study were obtained by breeding heterozygous adults (Fig. 1). We used a non-lethal birefringence assay to classify each larva as having either an affected or unaffected muscle phenotype several days prior to their escape response trials.

At 6 and 7 dpf, larvae were transferred one-at-a-time into an arena where escape responses were evoked with a brief electrical field pulse and recorded with high-speed videography. Each recording consisted of a 10 ms pre-stimulus period, during which time the larvae was stationary, a variable duration latency period (≈7 ms) that separated the stimulus and the initiation of tail movement, and the subsequent escape swimming activity. We analyzed the initial 60 ms of escape response swimming. Longer periods of swimming were more difficult to collect as there was an increased likelihood that the larvae would exit the camera’s field of view before recording was complete.

**Fig. 2:**
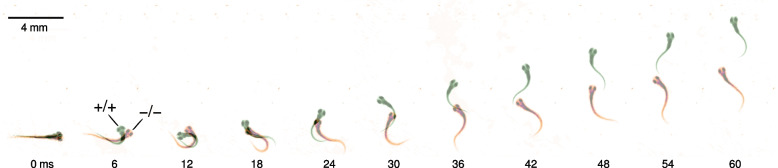
Representative escape responses. Shown are selected video frames (every 6th ms) from an escape response of a sapje mutant larva (−/−, pseudo-colored orange) and a wild-type sibling (+/+, pseudo-colored green). Numerals below the larvae indicate movement time in ms

Representative escape responses of a wild-type larvae and a *sapje* mutant are presented in Fig. 2. For brevity, we only present selected time points in this Figure but even at this reduced resolution it is apparent that the dystrophic larvae swam considerably slower than expected. By the end of the 60 ms movement period the dystrophic larvae trailed its wild-type sibling by about 4 mm or 1 body length (BL). A video montage of these two escape responses in their entirety is available as Supplementary Material 1.

### A DNN accurately identifies keypoints on escaping larvae

**Fig. 3:**
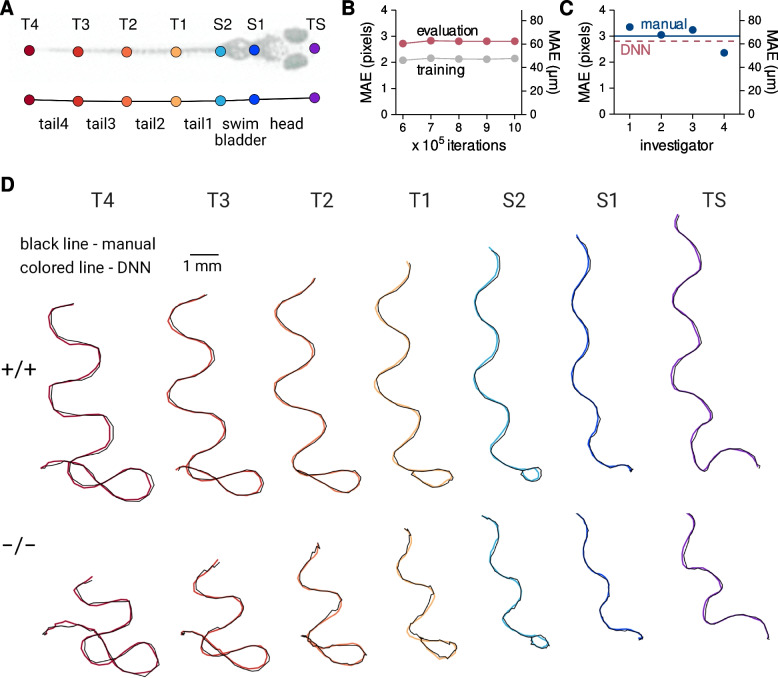
A deep neural network accurately estimates keypoints on escaping larvae. (**A**) Keypoints and the corresponding body segments used for modeling larvae. (**B**) The mean average Euclidean error (MAE) during training and evaluation of the deep learning-based neural network (DNN). (**C**) Four investigators manually annotated portions of an escape response video on two occasions. The MAE between their initial trial and the repeat trial are shown as blue points and the average MAE is indicated by the solid blue line. For reference, the MAE of the DNN is indicated by the red dashed line. (**D**) The most consistent annotator from Panel C manually annotated the two escape response videos shown in Figure 2. Keypoint escape response paths derived from the manual annotations and the DNN predictions are indicated by the black and colored lines, respectively

In order to quantify escape response movements, we modeled each escaping larva as six linked body segments (Fig. 3-A). We used the open-source pose estimation toolkit DeepLabCut [22, 23] to develop a deep-learning based neural network (DNN) for automated identification of the seven anatomical landmarks, or keypoints, that defined the body segments. The DNN was trained until it achieved a mean average Euclidean error (MAE) of 2.8 pixels which is equivalent to 64 μm under our experimental conditions (Fig. 3-B). This is similar to the test–retest accuracy of four human investigators tasked with manually identifying every third keypoint of a 60 ms escape response (Figs. 3-C). In addition, one investigator manually annotated the complete escape response of a wild-type and a mutant larvae (over 800 total keypoints). The MAE between this manual annotation and the DNN estimates of the same two escape responses was 4.2 pixels (Fig. 3-D).

### Modeling escape responses

**Fig. 4:**
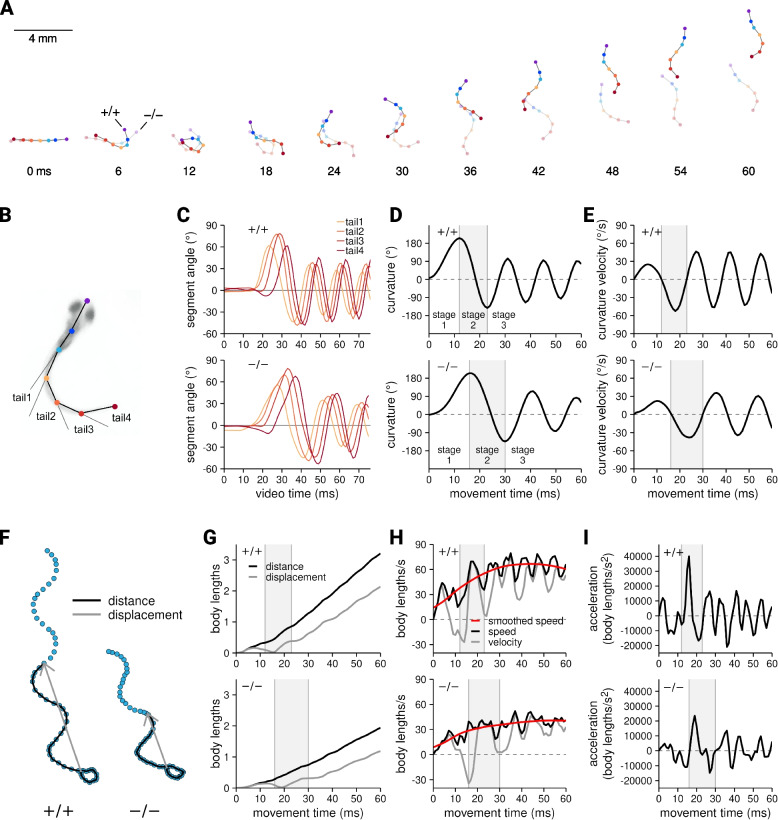
Quantification of escape response kinematics. (**A**) Linked segment models of the two escape responses originally shown in Figure 2. All subsequent data in this figure derived from these two models. (**B**) The coordinates of keypoints T1, T2, T3, and T4, were used to calculate the angle of each tail segment. (**C**) Tail segment angles vs. movement time. (**D**) Tail segment angle values were summed to yield tail curvature. Extremes in tail curvature was used to identify the transition from escape response stage 1 to 2 and from stage 2 to 3. (**E**) Tail curvature angular velocity vs. movement time. (**F**) Each point is the position of keypoint S2 (the putative center of mass) during the two escape responses. Distance and displacement at the 40th ms of movement are indicated by the solid black lines and gray vectors, respectively. (**G**) Distance and displacement vs. movement time. (**H**) Speed and velocity vs. movement time. Speed was smoothed for use in subsequent analyses. (**I**) Acceleration was derived from velocity. Linear kinematics were normalized to larval body length

The Cartesian coordinates output by the DNN were used to model each escaping larva as a linked-segment system moving through 2-dimensional space. Figure 4-A shows the two larvae previously presented in Fig. 2 now modeled as linked segments. A complete animated montage of these two models is available as Supplementary Material 2.

From the keypoint coordinates, we calculated 31 kinematic variables that mathematically described the angular kinematics of the propulsive tail (Fig. 4-B through -E) and the linear kinematics of the larva’s putative center of mass (Fig. 4-F through -I). These variables could be categorized as those describing overall performance (60 ms distance and displacement and the peak instantaneous speed and acceleration attained during that time period) and those that quantified movement within three distinct biomechanical stages that make up the escape response [28]. These stages were defined as the initiating C-start, a high amplitude body bend that draws the head and tail together (stage 1), a rapid reversal in tail curvature that generates an initial power stroke (stage 2), and a concluding burst of tail strokes in rapid succession (stage 3).

### Repeatability of escape responses

**Fig. 5:**
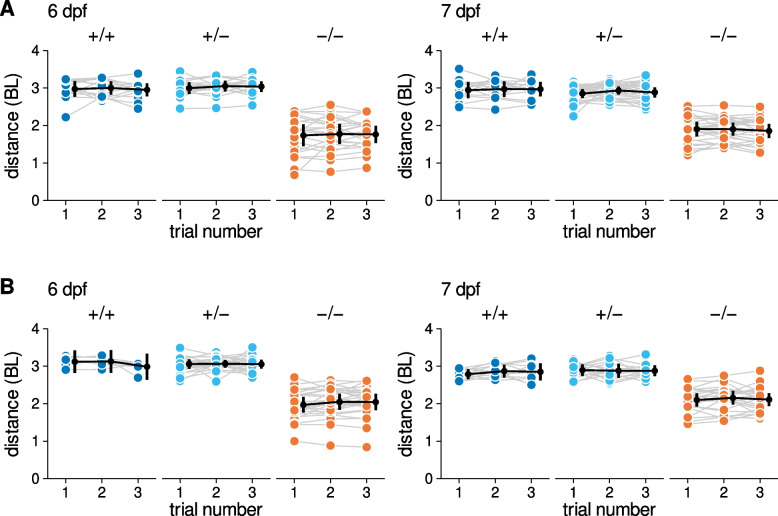
Escape response distance measurements are repeatable. (**A**) sapje larvae. (**B**) sapje-like larvae. Each symbol represents the distance covered during a single escape response trial. Trials from the same larvae are connected with lines. Black points are the trial mean and 99% confidence interval. Abbreviations: BL, body length. Data from 366 videos of sapje larvae (122 larvae, 3 trials/larvae) and 312 videos of sapje-like larvae (104 larvae, 3 trials/larvae)

We studied a total of 678 escape responses and for each trial the larvae was still moving at the 60 ms time point, although on some trials the larvae had slowed considerably (Supplementary Material 3, Figure S1). We addressed the repeatability of these repeated trials by focusing on the total distance covered in the response as this is a global marker of performance that encapsulates all other kinematic variables. Each larva completed three escape trials. In general, responses were consistent within a genotype, across days, and for both strains (Fig. 5). To quantify this apparent consistency, we calculated trial to trial repeatability coefficients [42]. For repeated distance measurements made on the same larva, trial-to-trial differences would be expected to be > 0.38 BL for +/+ larvae, > 0.38 BL for +/− larvae, and > 0.36 BL for −/− larvae in only 5% of cases. As a further check, we evaluated repeatability of peak instantaneous speed and observed comparable repeatability (Supplementary Material 3, Figure S2).

Based on the repeatability of these global indicators of overall escape performance, we collapsed trials into an average value. All data points and analyses going forward are based on a data set where each variable is represented by a single mean value per larva.

### Impaired escape response performance of dystrophic larvae

**Fig. 6:**
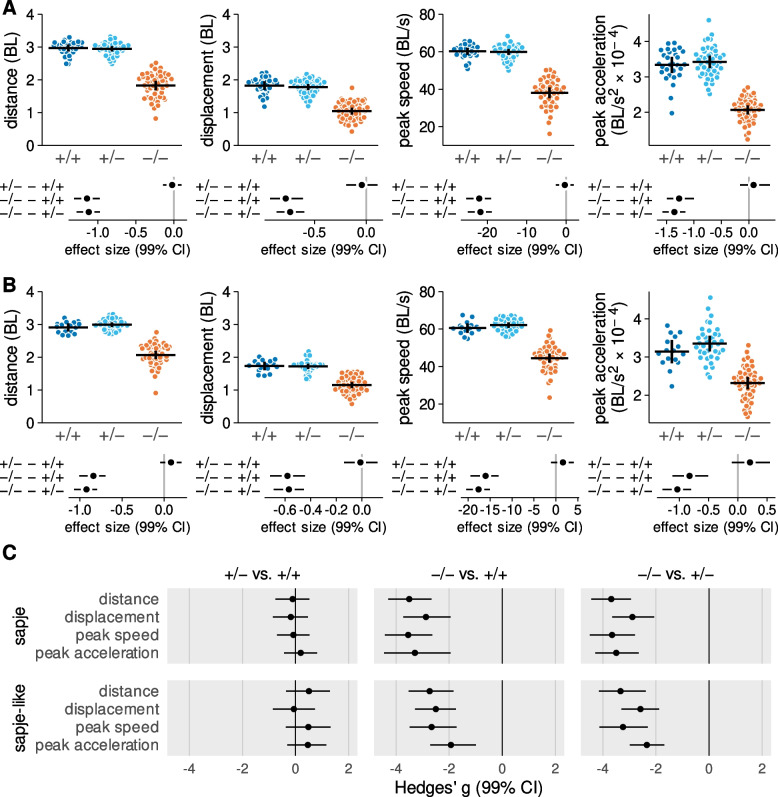
Escape response performance is impaired in dystrophic larvae. (**A**) sapje larvae. (**B**) sapje-like larvae. In Panels A and B each data point in the upper row of plots represents the mean value for a single larva (mean of three separate trials) with the horizontal bars indicating the genotype group mean and 99% confidence intervals. Below the dot plots are the bootstrapped effect sizes with 99% confidence intervals for each pairwise comparison (in the same units as the dependent variable). (**C**) Bootstrapped Hedges’ g with 99% confidence intervals for each genotype contrast. Units are standard deviations. Abbreviations: BL, body length. Data from 122 sapje strain larvae (29 +/+, 44 +/−, 49 −/−) and 104 sapje-like strain larvae (16 +/+, 37 +/−, 51 −/−)

We used escape response distance, final displacement, peak instantaneous speed, and peak acceleration as markers of overall escape response performance. The dot plots in the top row of Fig. 6-A and -B show performance values for each individual *sapje* and *sapje-like* larva along with the group means and 99% confidence intervals. The confidence intervals can be interpreted as the measurement error and are indicative of the high precision of the kinematic measurements.

Several kinematic variables did not appear to be normally distributed. To address this, we used a bootstrap procedure to re-sample the data set and calculate bootstrapped means and 99% confidence intervals for all between group comparisons [32]. These bootstrapped values are presented immediately below their respective dot plot in Fig. 6-A and -B. Based on the bootstrapped results, there was little evidence that escape response performance differed between wild-type homozygous and heterozygous larvae. In contrast, homozygous *sapje* and *sapje-like* mutants consistently under-performed their dystrophin-positive siblings across all four variables presented here. Taking escape response distance as an example, our best estimate is that *sapje* mutants covered 1.1 BL less distance after 60 ms of escape response swimming compared to the dystrophin-positive larvae, with plausible values ranging from a deficit of 0.98 to a deficit of 1.3 BL (plausible values are defined by the 99% confidence intervals). Because a between group difference of zero is not a plausible value, the differences between mutants and the wild-type groups are statistically significant (at *p* < 0.01 or less).

Mutants also displaced themselves less than expected during an escape response but effect sizes for displacement were about one-third less than noted for distance. Comparisons between distance and displacement are possible because both are expressed in the same units. In order to facilitate comparisons of effect sizes across variables of different measurement units, we calculated bootstrapped Hedges’s *g* [43], a standardized effect size that is expressed in units of standard deviations (SD) (Fig. 6-C). Bootstrapped effect sizes of 3.5 SD distinguished distance, peak instantaneous speed, and peak acceleration of *sapje* mutants from their wild-type siblings, with the effect size for displacement slightly less at 2.8 SD. For *sapje-like*, effect sizes were somewhat less than observed in *sapje* larvae but were still substantial, ranging from 1.9 to 3.3 SD’s.

### Biomechanical mechanisms underlying impaired mobility

**Fig. 7:**
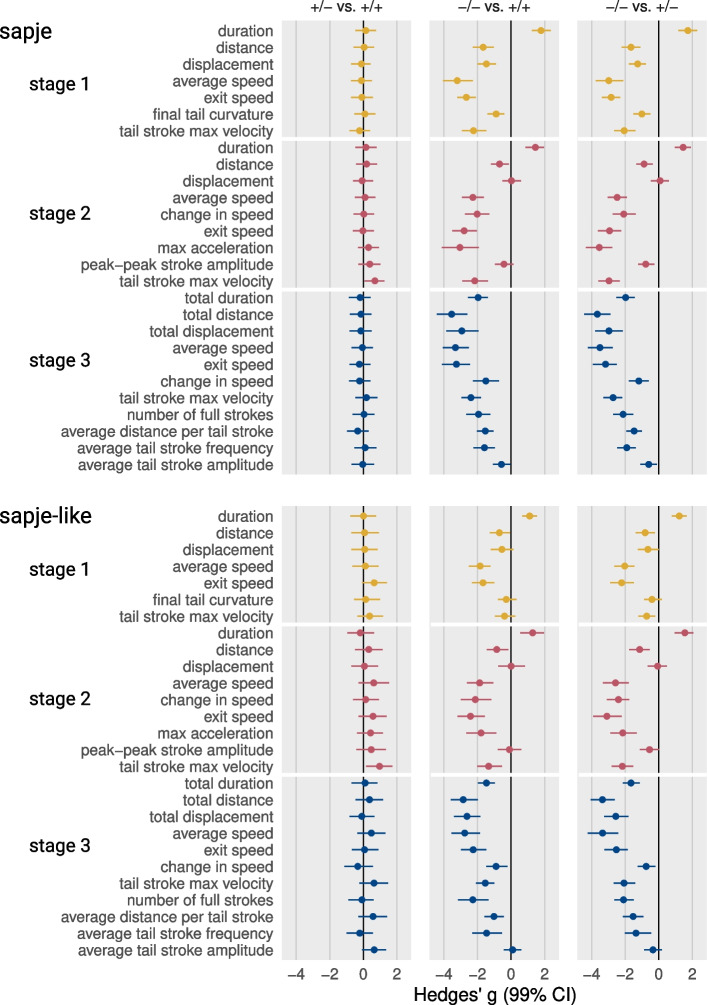
Kinematic profiles reveal mechanisms underlying impaired swimming of dystrophic larvae. Points are bootstrapped Hedges’ g with 99% confidence intervals. Units are in standard deviations. Plot interpretation and sample sizes same as Figure 6-C. Abbreviations: max, maximum

To identify reasons why escape response swimming performance was impaired in dystrophic larvae we evaluated 27 kinematic properties that quantified center of mass movement and tail mechanics. Figure 7 presents Hedges’ *g* for these kinematic variables classified by escape response stage for both strains of larvae. The left most column of plots indicates that there is little difference in the escape response kinematics of wild-type homozygous and heterozygous larvae. The second and third columns reveal that *sapje* and *sapje-like* mutants show a very similar escape response kinematic profile. However, their profiles differ markedly from the profile of their respective wild-type siblings. Effect sizes between mutant and wild-type larvae exceeded 2 SD’s for many variables and 3 SD’s in some cases.

During fast swimming, zebrafish larvae adduct their pectoral fins so that propulsive forces are generated solely by the movements of the tail [44]. Therefore, the speed a fast swimming larva can attain is a function of the amplitude and frequency of their tail strokes. Figure 7 provides insight into how these parameters were impacted by the absence of dystrophin. Peak to peak tail amplitude appeared to be only mildly affected, if affected at all, in dystrophin mutants. For instance, there were relatively small differences between mutant and wild-type larvae in the final tail curvature of stage 1, the change in tail curvature during the power stroke of stage 2, and the average amplitude of tail strokes in stage 3. In contrast, mutants showed a substantial slowing in the maximum angular velocity of tail strokes across all three stages and a slowing of stage 3 average tail stroke frequency.

A slower oscillating tail, even when coupled with a roughly unaffected tail stoke amplitude, will produce less propulsive power to move the larva forward. Thus, the slower stage 2 power stroke of mutants generated less acceleration (stage 2 max acceleration) which slowed the larva’s speed as it began stage 3 burst swimming (stage 2 exit speed). Then in stage 3, the distance that each tail stroke propelled the dystrophic larvae (stage 3 average distance/tail stroke), the average speed of the larva (stage 3 average speed) and its speed at the 60 ms time point (stage 3 exit speed) were all reduced, contributing to a reduction in the distance the larva covered in stage 3 (stage 3 distance).

In addition to impacting propulsion, slower tail angular velocities and stroke frequencies affected several other aspects of the escape response that exacerbated the decline in performance. Slower tail strokes prolonged stages 1 and 2 which in turn left less time for stage 3 swimming. Because stage 3 swimming time was reduced, dystrophic larvae completed fewer stage 3 tail strokes (stage 3 average number of tail strokes) than wild-type larvae. These changes were critical as the majority of the distance covered in 60 ms of escape response swimming (see Fig. 4-G) normally takes place in stage 3.

### Kinematics are predictive of the dystrophic genotype

**Fig. 8:**
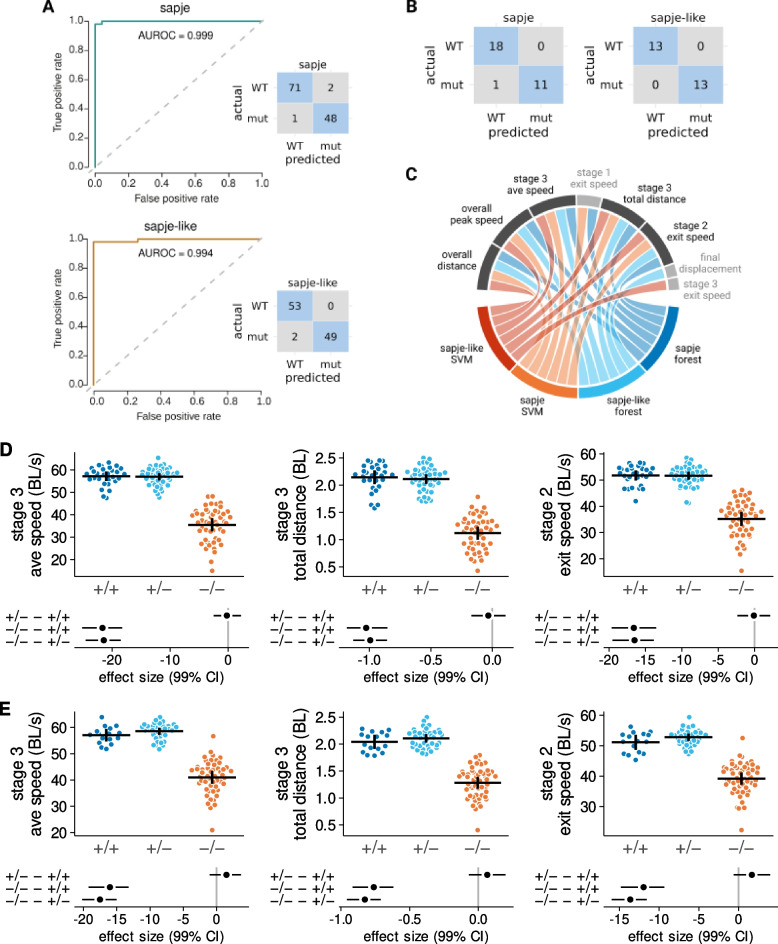
Swimming kinematics are highly predictive of larval genotype. (**A**) Random forest model area under receiver operating characteristic curve and confusion matrix results for sapje and sapje-like larvae. (**B**) Support vector machine (SVM) model confusion matrices for sapje and sapje-like larvae. (**C**) Chord plot of the 6 best predictors from the forest and SVM classification models. Five kinematic variables were identified that were common to all four of the strain by predictor model combinations. Descriptions of two of these variables (overall distance and overall peak speed) have been presented in Figure 6. The remaining variables are presented in detail here for (**D**) sapje larvae, and (**E**) sapje-like larvae. Interpretation and samples sizes of Panel D and E same as in Figure 6-A and -B, respectively. Abbreviations: mut, mutant; AUROC, area under receiver operating characteristic curve

We next asked whether kinematic variables could be used to predict larval genotype. A random forest model, using all 31 kinematic variables, was developed to differentiate between dystrophic (−/−) and wild-type larvae (+/+ and +/−) of both the *sapje* and the *sapje-like* strains. The model discriminated between mutant and wild-type larvae with high accuracy, attaining AUROC (area under the receiver operating characteristic curve) values of 0.994 to 0.999 and error rates of ≤ 2.5% (Fig. 8-A). We confirmed these results using a SVM predictive model. The SVM analysis was equally accurate, differentiating mutant from wild-type larvae with an error rate ≤ 3% (Fig. 8-B).

In order to identify which of the 31 kinematic variables were most predictive, we created a chord plot based on the top six predictors from each of the four strain x predictive model combinations (Fig. 8-C). This plot revealed that five kinematic variables were common to each combination: overall distance and peak instantaneous speed (previously discussed in Fig. 6), the average speed during stage 3 burst swimming and the distance covered in that stage, and the speed with which larvae exited stage 2 following the power stroke (Fig. 8-D and -E). These five variables capture key kinematic features of the escape response that distinguish dystrophin-deficient mutants from wild-type larvae.

### Kinematics predict genotype as well as measurements of muscle contractility

**Fig. 9:**
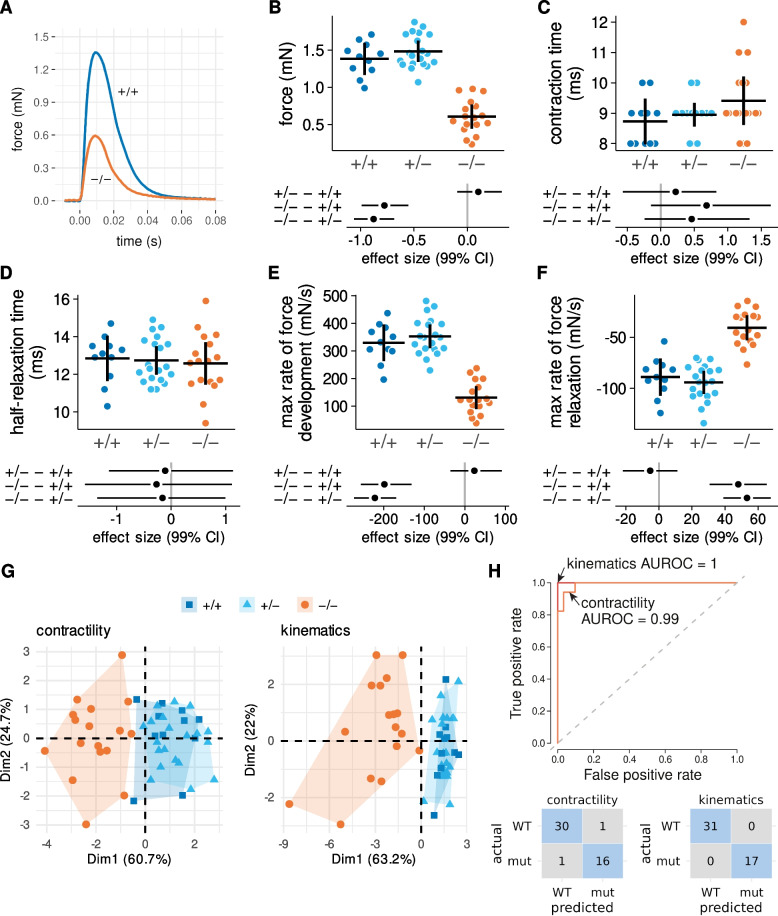
Tail angular kinematics and tail muscle contractility predict genotype with similar accuracy. (**A**) Representative twitch force records from tail muscle preparations of a wild-type and sapje mutant. Twitches were used to determine (**B**) peak force, (**C**) contraction time, (**D**) half-relaxation time, (**E**) maximum rate of force development, and (**F**) maximum rate of force relaxation. Sample sizes for contractility measurements: 11 +/+, 20 +/−, 17 −/− sapje larvae. (**G**) Biplot of principle components 1 (Dim1) and 2 (Dim2) for these five contractility variables and nine variables from Figure 7 describing tail kinematics (see text for details). (**H**) Random forest classification models for the prediction of genotype using contractile or kinematic variables. Abbreviations: mut, mutant; AUROC, area under receiver operating characteristic curve

Dystrophic zebrafish larvae are characterized by weak tail muscles [30, 45] and we asked if this weakness was consistent with their impaired escape response swimming. We confirmed tail muscle weakness in sub-groups of mutant and wild-type *sapje* larvae after we had collected videos of their escape response performance. In vitro tail muscle preparations indicated that preparations from mutant *sapje* larvae generated only 42% of the twitch force observed for preparations from wild-type larvae (Fig. 9-A and -B). Twitch contraction time and half-relaxation time were similar across genotypes but the rates of twitch tension development and relaxation were reduced for the *sapje* mutants (Fig. 9-C to -F).

We tested how well these five measures of tail muscle contractile function differentiated between genotypes. We did the same for nine kinematic variables describing the motion of the propulsive tail (from stage 1: duration (which is the same as tail stroke duration), final tail curvature, and tail stroke maximum angular velocity; from stage 2: tail stroke duration, tail stroke amplitude, and tail stroke maximal angular velocity; from stage 3: average tail stroke frequency, average tail stroke amplitude, and tail stroke maximal angular velocity). Principal component analyses indicated that the first two dimensions accounted for 80% of the total variability between genotypes regardless of whether the analysis was conducted with contractile or kinematic variables (Fig. 9-G). Furthermore, both approaches appeared equally capable of segregating dystrophic from wild-type larvae. Finally, a random forest model using muscle contractility as a predictor of genotype misclassified 2 out of 48 larvae (Fig. 9-H). The same model using the kinematic variables as predictors of genotype correctly classified all larvae.

## Discussion

The startle or escape response is one of the most well studied swimming behaviors of zebrafish larvae [14, 28, 46]. The response is mediated by reticulospinal neurons that descend bilaterally along the spinal cord to synapse with interneurons and motorneurons of the trunk and tail musculature. The relatively simplicity of this neural circuit, its all-or-none nature and accessibility to experimental manipulations, and the availability of methods for high speed recording and quantification of the subsequent motor output have made experimentally evoked escape responses an important tool for understanding vertebrate sensimotor integration [12, 14, 28, 47, 48].

Visual examination of touch-evoked escape responses has proven to be a valuable, although somewhat subjective, approach for identifying mutants that model human muscle diseases [19, 49–52]. Only rarely have these models been evaluated using the high sampling rates that are common in studies of sensimotor integration [53]. Because escape responses have a key role in the development of therapeutic interventions targeting skeletal muscle diseases [40], we aimed to greatly expand the breadth and depth of information that could be obtained by this approach. We utilized the high sampling rates typical of sensimotor studies and coupled this with machine learning and kinematic analysis to quantify the mobility deficits of two zebrafish models of Duchenne muscular dystrophy.

We observed large effect size deficits in escape response swimming of dystrophin mutants. Following the first 60 ms of escape response movement, mutants trailed their wild-type siblings by an average of over 1 body length. By examining the mechanics of the propulsive tail, we found that the absence of dystrophin had little or only very modest effects on peak-to-peak tail stroke amplitude during an escape. In contrast, there was a substantial slowing in the frequency and angular velocity of tail strokes. Slower tail strokes would be expected to have a direct effect on the generation of power for propulsion, consistent with the reduced acceleration produced by the stage 2 power stroke and the subsequent slower swimming speed of mutant larvae. A slowing of tail strokes also had a cascading effect on temporal aspects of our 60 ms escape responses, such as reducing the time available for critical stage 3 burst swimming.

In contrast to our findings, it was previously reported that the peak acceleration attained during touch-evoked escape responses did not differ between *sapje* and wild-type larvae [52]. Importantly, this previous work used manually dechorinated 2 dpf larvae vs. the 6–7 dpf larvae used here. Birefringence assays indicate that disruption of the axial musculature does not occur in *sapje* until 3–4 dpf, which is after larvae have hatched and started to spontaneously swim about [49]. Considering the previous 2 dpf results alongside the current 6–7 dpf data suggests that locomotor deficits in dystrophin-deficient larvae are a progressive event triggered by normal muscular activity, similar to how the disease manifests in human patients.

While a slowing in the oscillating motion of the tail seems to be the primary mechanism underlying the impaired mobility of dystrophin mutants, we found no evidence that the intrinsic rate of muscle contraction was altered in isolated tail muscle preparations from mutant *sapje* larvae. For example, we detected no difference in twitch contraction time between mutant and wild-type tail muscle preparations. In the absence of evidence for a slowing in intrinsic shortening, we propose that the slowing of the tail stroke rate is a secondary consequence of the muscle weakness that characterizes dystrophin mutants.

We identified five kinematic variables that were highly predictive of the dystrophic genotype. These predictors appear quite robust as all five were identified by two independent prediction models applied to two different strains of dystrophic larvae. These variables could be valuable biomarkers of the dystrophic genotype in future studies. All five were linear kinematic measurements: the overall distance the larvae swam in 60 ms, its peak instantaneous speed during that time, the distance covered and average speed during stage 3 swimming, and the stage 2 exit speed. It is important to note that because zebrafish are undulatory swimmers, high sampling rates are required in order to quantify the distance they cover during a swimming bout. Measurements made from where a larvae starts moving to where it stops defines their displacement and displacement measurements are not as robust as the candidate biomarkers identified here.

Because of the precise nature of these biomarkers, they are also statistically powerful. To illustrate, we used the R package ’pwr’ [54] and the present data to calculate theoretical power curves (Supplementary Material 3, Figure S3). This analysis indicated that if a treatment improves escape response distance of a group of dystrophin mutant larvae by a modest 20%, this difference would be detectable (80% power at *p* = 0.05) from an untreated group of mutants with sample sizes of about 15 larvae/group. Because our kinematic approach is non-lethal, studying the same larvae before and after treatment may be possible. In this paired experimental design, the required sample size falls to less than 10 larvae. These sample sizes are considerably less than the hundreds of larvae per condition that are sometimes used when assessing spontaneous swimming activity [20].

Tail kinematics and muscle contractility accounted for almost identical amounts of variability in our data set and both approaches were excellent predictors of larval genotype. These findings support the use of kinematic measurements as a proxy for direct in vitro measures of tail muscle contractility. Compared to in vitro measurements, kinematic analysis presents a number of advantages: it is non-contact, non-lethal, and technically less demanding. Its non-lethal nature is particularly noteworthy as it opens up new possibilities in experimental design, such as serial measurements conducted on the same larvae.

The kinematic approach outlined here has limitations. First, as currently configured, it is not a high throughput approach. This is due to biological factors such as the recovery time between trials to prevent fatigue and habituation, and technical factors which stem mainly from the small size of larvae and their tendency to spontaneously swim out of the cameras field of view. The present approach is therefore not amenable to screening hundreds of compounds. However, once a screen has narrowed the field to a few candidate therapeutics, the method described here could serve as a way to more thoroughly evaluate these compounds, particularly their effects on larval physiology, before advancing candidates to more resource-intensive mammalian models. The present approach could also be useful in evaluating chemical variants of a promising candidate compound.

Advances in machine vision and learning may present solutions to the low-throughput nature of the present approach. For instance, 13 unique swimming patterns, including short-latency C-starts which are equivalent to the stage 1 C-start described here, were recently identified in zebrafish larvae using an unsupervised machine learning approach [29].

A second limitation of the present approach concerns potential interactions between escape response fiber type recruitment and fiber-type specific mutations. The musculature of the larval tail consists of a thin, superficial layer of slow contracting red fibers surrounding a vastly larger volume of fast, white fibers [55, 56]. In zebrafish larvae, motor neurons are recruited by the size principal, i.e. from slow to fast as the intensity of activity increases [57]. Even though both slow and fast fibers are recruited during a larval escape response [58], because of the sheer volume and faster contraction speed of the white fibers, the fast motor unit pool generates the propulsive power for movement [18]. This is an advantage when using escape responses to evaluate mutations that impact all fiber types or fast fibers exclusively but presents a limitation when studying disorders that impact the small pool of slow, oxidative fibers. Preliminary data from our laboratory indicates that escape response kinematics may fail to identify larvae with mutations impacting mitochondrial function [59] or that target proteins expressed exclusively in slow muscle fibers [60]. Investigators may need to consider other approaches for evaluating mobility when studying fish with these types of mutations.

Finally, the majority of what is known about escape responses has been obtained by monitoring larvae in shallow arenas and we opted to continue with this two-dimensional approach. However, in deep arenas escape responses occur in three dimensions, with wild-type larvae showing changes in pitch of up to 30 degrees [61]. Future studies will be required to determine whether the additional information gained by three-dimensional modeling is balanced by its increased technical and computational demands.

## Conclusions

We have developed high temporal and spatial resolution kinematic models of escaping zebrafish larvae. From the models we identified repeatable, precise, physiologically relevant kinematic variables that reveal large effect size differences in the swimming performance of dystrophin-deficient and wild-type larvae. The approach described here opens new possibilities for future studies using zebrafish larvae to investigate DMD, other muscle diseases, and movement disorders in general.

## Supplementary Information

Supplementary Material 1.

Supplementary Material 2.

Supplementary Material 3.

## Data Availability

The datasets generated and/or analysed during the current study are available in the Harvard Dataverse repository, https://dataverse.harvard.edu/dataverse/sapje, under the following URLs: 10.7910/DVN/N2JOEO; 10.7910/DVN/MT4Z2G; 10.7910/DVN/WN3ZYH.

## Abbreviations

DMD: Duchenne muscular dystrophy
ER: Escape response
DNN: Deep-learning neural network
MAE: Mean average Euclidean error
BL: Body length
COM: Center of mass
LOESS: Locally estimated scatterplot smoothing
SVM: Support vector machine
ms: Millisecond
