# Supplementary figures and images for: Identifying kinematic biomarkers of the dystrophic phenotype in a zebrafish model of Duchenne muscular dystrophy
Source: Skelet Muscle. 2025 Jun 20;15:17. doi: 10.1186/s13395-025-00382-6 (PMC12180146; doi:10.1186/s13395-025-00382-6)

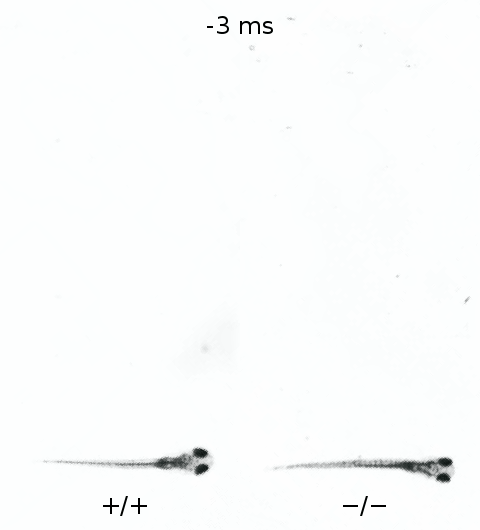

Supplement: Supplementary file 1 — Supplementary Material 1. [file 13395_2025_382_MOESM1_ESM.gif]

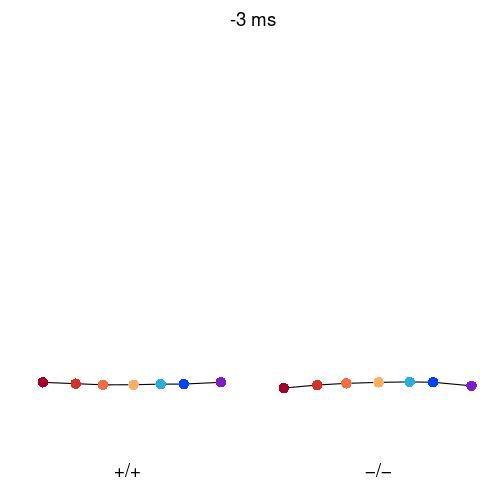

Supplement: Supplementary file 2 — Supplementary Material 2. [file 13395_2025_382_MOESM2_ESM.gif]
