# Supplementary material for: Identifying kinematic biomarkers of the dystrophic phenotype in a zebrafish model of Duchenne muscular dystrophy
Source: Skelet Muscle. 2025 Jun 20;15:17. doi: 10.1186/s13395-025-00382-6 (PMC12180146; doi:10.1186/s13395-025-00382-6)
Supplement: Supplementary file 3 — Supplementary Material 3. [file 13395_2025_382_MOESM3_ESM.pdf]

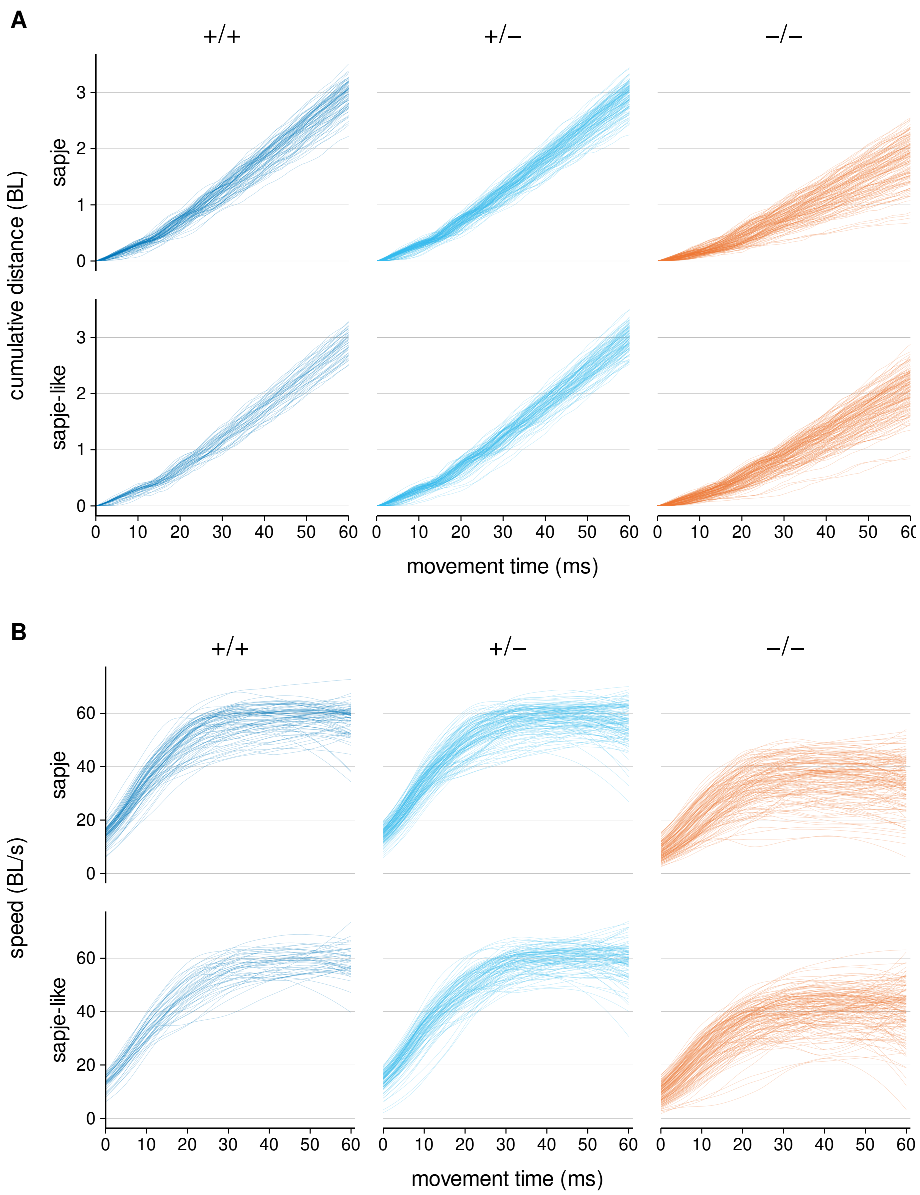

Figure S1. Frame-by-frame (ms by ms) evaluation of escape responses. Each line represents a single escape response trial (366 trials for *sapje*, 312 trials for *sapje-like*). **(A)** Cumulative distance. **(B)** Instantaneous speed. Instantaneous speed response smoothed as illustrated in Figure 4-H. Abbreviations: BL, body lengths.

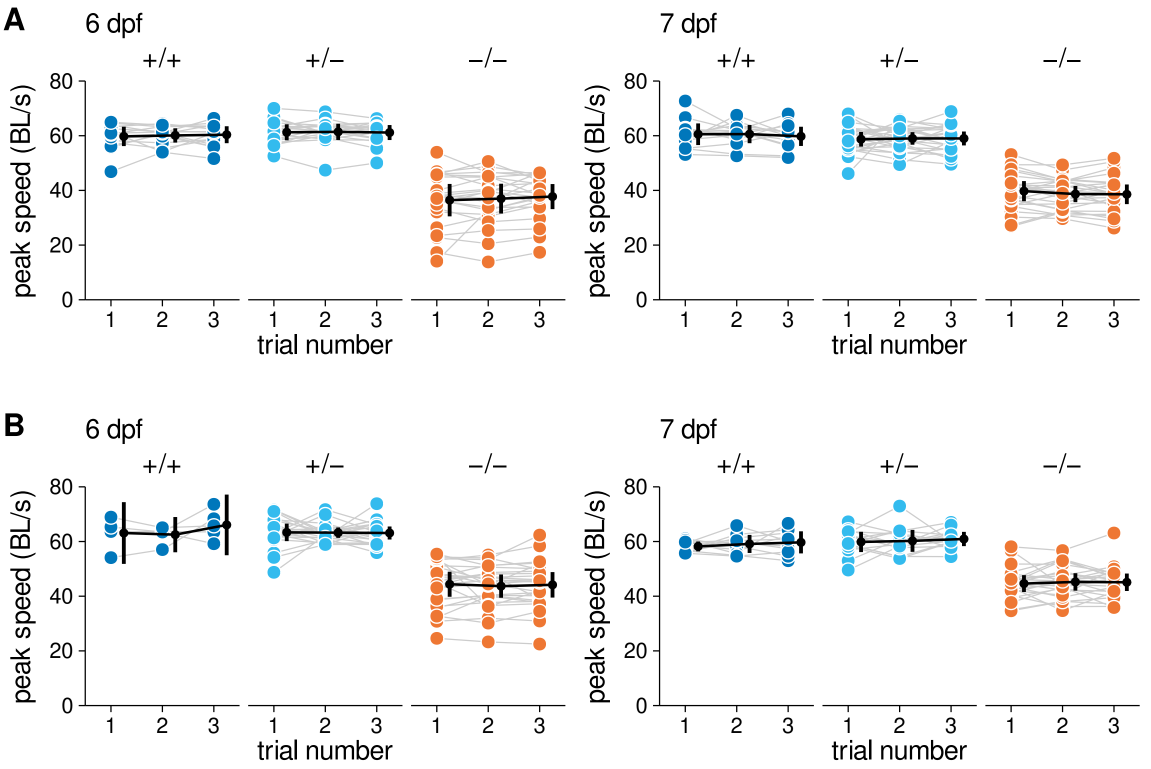

Figure S2. Repeatability of peak instantaneous speed. Lines and symbols same as in Figure 5. In only 5% of cases, would trial-to-trial differences be expected to be > 7 BL/s for a +/+ larvae, > 8 BL/s for a +/− larvae, and > 7 BL/s for a −/− larvae. Abbreviations: BL, body length. Data from the same larvae shown in Figure 5.

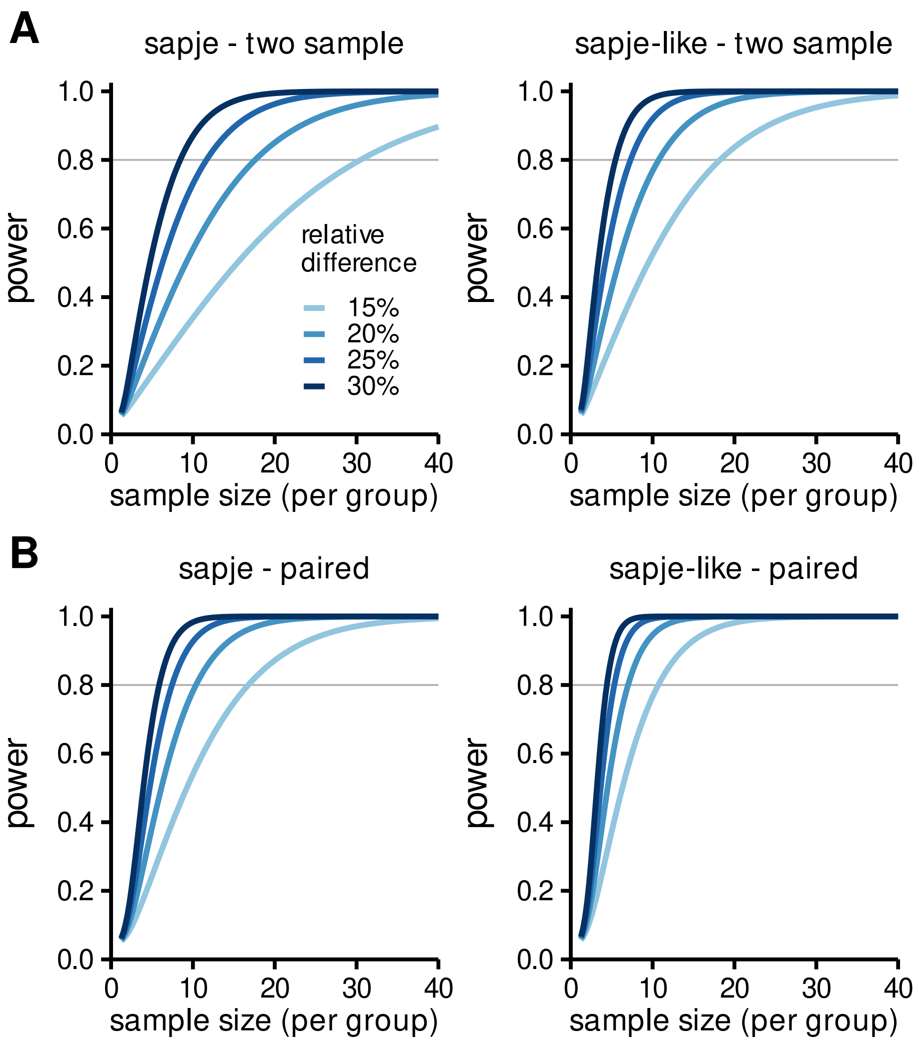

Figure S3. Estimated statistical power. Estimates are based on detecting relative differences in escaped response distance ranging from 15% to 30% using a two sided t-test at p = 0.05. **A** Power curves for a two-sample design. **B** Power curves for a paired design. Power calculations were based on the mean and SD of the *sapje* and *sapje-like* mutant larvae in this project and the assumption that those SD's do not change as a result of treatment.
